# Anti-Oxidative Stress Activity of *Stachys lavandulifolia* Aqueous Extract in Human

**Published:** 2013-02-20

**Authors:** Kobra Rahzani, Ali Akbar Malekirad, Akbar Zeraatpishe, Naser Hosseini, Seyed Mohammad Reza Seify, Mohammad Abdollahi

**Affiliations:** 1. Department of Nursing, Arak University of Medical Sciences, Arak,Iran; 2. Department of Biology, Payame Noor University, Iran; 3. Faculty of Pharmacy, Pharmaceutical Sciences Research Center, Tehran University of Medical Sciences, Tehran, Iran; 4. Department of Biology, Jiroft Branch, Islamic Azad University, Jiroft, Iran; 5. Department of Medicinal Plants, Faculty of Agriculture and Natural Resources, Arak University, Arak, Iran; 6. Department of Agriculture, Payame Noor University, Iran

**Keywords:** Antioxidant Effects, Clinical Trial, Medicinal Plants, Oxidative Stress, Stachys

## Abstract

Medicinal plants are presumed to be natural sources of antioxidants that protect organisms from oxidative stresses. The present investigation aims to study the anti-oxidative stress activity of the *Stachys lavandulifolia (S. lavandulifolia)* plant. This trial was conducted on 26 healthy human subjects. The study was done in a before after fashion. The included subjects were asked to consume the prepared infusion from 3 g aerial parts of *S. lavandulifolia* on a daily basis. Doses were administered in every morning and evening for 14 days. At the beginning and the end of the study, blood samples were acquired to determine the level of cellular lipid peroxidation and the total content of serum antioxidants. Biomarkers analyzed from samples obtained before start of treatment and 14 days post treatment, were subjected to paired t test analysis. Total blood antioxidants increased and reached from 2.3 ± 0.84 µmol/ml to 3.3 ± 0.54 µmol/ml. The lipid peroxidation reduced and reached from 8.38 ± 1.78 to 11.6 ± 2.64 nmol/ml. The results suggest that *S. lavandulifolia* possesses marked anti-oxidative stress activity and it can be useful as a supplement in the management of diseases related to oxidative stress (Registration Number: IRCT2013012210003N2).

*Stachys lavandulifolia (S. lavandulifolia)* is an aromatic plant belonging to the Labiatae family. In Iran, *S. lavandulifoliais* is widely found in Azerbaijan, Golestan, Khorasan, Mazandaran, and Tehran (1). The plant contains large amounts of flavonoids (2). The antibacterial, antitoxic, antihepatitis, hypotensive, and antianxiety properties of different Stachys species’ extracts have previously been documented in a line of pharmacological studies. In Iranian folk medicine, the aerial parts of this plant have been used as an analgesic and an antiinflammatory (3).

In normal human life, environmental pollutants, stress, and different diseases cause oxidative stress that results in the damage of biomolecules such as proteins, lipids, amino acids and nucleic acids. Therefore, to prevent cell injuries the body defends itself by means of enzymatic or non-enzymatic antioxidants. Enzymes such as superoxide dismutase, glutathione peroxidase, and catalase and nonenzymatic ones such as vitamin E, carotenoids, ascorbic acid, and bilirubin play a role (4-8). Factors such as environmental pollutants disturb the balance between formation and elimination of free radicals and, as a result, oxidative stresses occur that are considered to cause many diseases such as cancers and diabetes as well as contributing to the aging process (9-14).

In previous studies the antioxidant potentiality of the aerial components of *S. lavandulifolia* has been reported (15-18). The present study aimed to investigate the effect of an aqueous extract of *S. lavandulifolia* on the oxidative stress status of healthy people.

Chemicals used for oxidative stress analyses were purchased from Merck Chemical Co. (Germany) unless otherwise stated. In this study, a clinical investigation was performed on 26 individuals (10 men, 16 women), aged 16 to 55 years, who were randomly selected from the Young Research Center of Arak after they were informed about the study and their consent was acquired prior to their enrollment into the study. The study was conducted in complete accordance with the National Code of Ethics and approved by the Ethics Committee of the Pharmaceutical Sciences Research Center at Tehran University of Medical Sciences.

All participants were healthy individuals with no histories of smoking, alcohol, drug, or antioxidant use. A separate questionnaire was prepared for each subject and they were asked to record their diet during the experiment. A before after trial was done. A five ml of blood sample was taken at the beginning and the end of the study from each individual assigned to consume 3 g of the *S. lavandulifolia* infusion twice daily at the morning and the evening. Afterwards, blood samples were centrifuged and their serum separated and analyzed for oxidative stress parameters.

The thiobarbituric acid (TBA) method was used to measure lipid peroxidation. As a result of free radical attack, different aldehydes such as malondialdehyde (MDA) are produced from lipids that react with TBA under acidic conditions and high temperatures. The resultant complex has a maximum absorbance at 532 nm. To evaluate lipid peroxidation, serum proteins were initially precipitated with the addition of 2.5 ml TBA to 0.5 ml serum and kept for 10 minutes at room temperature. The mixture was subsequently centrifuged at 3000 g for 10 minutes, the supernatant removed, and the precipitate washed with 0.5 M sulfuric acid. After wards, 2.5 ml of 0.5 M sulfuric acid and 3 ml of a 0.2% TBA solution were added to each tube. After preparation of the 3 ml 0.2% TBA solution and 2.5 ml of each standard, all samples other than these solutions were incubated in a 100℃ water bath for 30 minutes. Subsequently, 4 ml n-buthanol was added to each cold tube and mixed well by vigorous vortexing. Finally, the mixture was centrifuged at 3500 g for 10 minutes and the absorbance of the supernatant recorded at 532 nm (19).

To measure total antioxidant power, we used the ferric reducing ability (FRA) method which is based on the ability of serum to reduce Fe3+ cations to Fe^2+^ in the presence of 2, 4, 6-tripyridyl- striazine (TPTZ). The Fe^2+^ –TPTZ complex has a maximum absorbance of 593 nm. To determine serum antioxidants, 25 ml phosphate buffer was mixed with 2.5 ml ferric chloride and 2.5 ml TPTZ to produce the FRA solution, which should be prepared fresh and immediately used. Of the prepared FRA solution, 1.5 ml was added to each tube and the tubes were incubated at 37℃ for 5 minutes. Next, 50 µl of each serum sample was added to each tube and kept at 37℃ for 5 minutes. Finally, the absorbances of all tubes were separately recorded at 593 nm and their antioxidant content determined according to the obtained standard curve (20). All data were tested by paired t test using Stats Direct 2.7.9.

The paired t test showed significant differences between the total content of the serum antioxidant ability before and after treatment of the subjects with the aerial parts of *S. lavandulifolia* (p=0.001). Following treatment, FRA increased from 2.3 ± 0.84 µmol/ml to 3.3 ± 0.54 µmol/ml. Similarly, the treatment had a significant effect (p=0.0001) on the level of cellular lipid peroxidation, which decreased after treatment from 11.6 ± 2.64 nmol/ml to 8.38 ± 1.78 nmol/ml (Table 1). The relation between age, gender, and oxidative stress parameters was not significant according to the results of the Pearson correlation analysis.

**Table 1:** The effect of aqueous infusion of Stachys Iavandulifolia on blood oxidative stressparameters


Oxidative stress parameter	Before treatment	After treatment	P value

Total antioxidant power	2.3 ± 0.84	3.3 ± 0.54	0.001
Lipid peroxidation	11.6 ± 2.64	8.38 ± 1.78	0.0001


Taking the results collectively, treatment with the aerial parts of *S. lavandulifolia* led to a considerable reduction in oxidative stress. In support of this finding, the antioxidant potential of numerous *Stachys* species, including *S. lavandulifolia*, has been reported by other investigators (2, 15, 21, 22), but this is the first trial in human. Oxidative stress plays a major role in the pathogenesis or progress of many debilitating diseases or conditions in humans such as osteoporosis (23), diabetes (11, 24-28), islet transplantation (29), inflammatory bowel diseases (12, 30), preeclampsia (31), pancreatitis (32), metaltoxicity (6, 33), or pesticide poisoning (5). The results of the present study are optimistic and show marked antioxidant activity of the *S.lavandulifolia* extract in healthy individuals.

Therefore, *S. lavandulifolia* can be used as a supplement to protect individuals from oxidative stresses in the above-mentioned diseases.
